# Designing small molecules to target cryptic pockets yields both positive and negative allosteric modulators

**DOI:** 10.1371/journal.pone.0178678

**Published:** 2017-06-01

**Authors:** Kathryn M. Hart, Katelyn E. Moeder, Chris M. W. Ho, Maxwell I. Zimmerman, Thomas E. Frederick, Gregory R. Bowman

**Affiliations:** 1 Department of Biochemistry & Molecular Biophysics, Washington University School of Medicine, St. Louis, Missouri, United States of America; 2 Department of Biomedical Engineering, and Center for Biological Systems Engineering, Washington University in St. Louis, St. Louis, Missouri, United States of America; Universidad de Santiago de Compostela, SPAIN

## Abstract

Allosteric drugs, which bind to proteins in regions other than their main ligand-binding or active sites, make it possible to target proteins considered “undruggable” and to develop new therapies that circumvent existing resistance. Despite growing interest in allosteric drug discovery, rational design is limited by a lack of sufficient structural information about alternative binding sites in proteins. Previously, we used Markov State Models (MSMs) to identify such “cryptic pockets,” and here we describe a method for identifying compounds that bind in these cryptic pockets and modulate enzyme activity. Experimental tests validate our approach by revealing both an inhibitor and two activators of TEM β-lactamase (TEM). To identify hits, a library of compounds is first virtually screened against either the crystal structure of a known cryptic pocket or an ensemble of structures containing the same cryptic pocket that is extracted from an MSM. Hit compounds are then screened experimentally and characterized kinetically in individual assays. We identify three hits, one inhibitor and two activators, demonstrating that screening for binding to allosteric sites can result in both positive and negative modulation. The hit compounds have modest effects on TEM activity, but all have higher affinities than previously identified inhibitors, which bind the same cryptic pocket but were found, by chance, via a computational screen targeting the active site. Site-directed mutagenesis of key contact residues predicted by the docking models is used to confirm that the compounds bind in the cryptic pocket as intended. Because hit compounds are identified from docking against both the crystal structure and structures from the MSM, this platform should prove suitable for many proteins, particularly targets whose crystal structures lack obvious druggable pockets, and for identifying both inhibitory and activating small-molecule modulators.

## Introduction

Rational drug design based on a single protein structure captured, for instance, by x-ray crystallography typically focuses on molecules that bind to and sterically block a key functional site. Therefore, this approach is inapplicable to proteins that lack obvious druggable pockets or scenarios where activation, rather than inhibition, is desired. However, proteins are not static objects. They are ensembles of structures populated at equilibrium according to each state’s thermodynamic stability. It is possible to access many of the alternative structures a protein adopts by methods such as NMR [1] or molecular dynamics simulations [2]. Druggable pockets that appear in these alternate structures, called cryptic pockets, present the opportunity to design allosteric drugs, which bind to proteins in regions other than their main ligand-binding or active sites and are known to have distinct benefits over drugs targeting active sites [3]. For example, there is good reason to believe that activator compounds would prove efficacious against diseases as diverse as cancer [4], liver disease [5] and diabetes [6]. While there are examples of high-throughput experimental screens that have serendipitously identified compounds that bind cryptic pockets [7] and screens designed specifically for finding allosteric modulators [8], our goal is to develop a structure-based approach to rationally target cryptic pockets in proteins for drug design.

As a proof of principle, we chose to target TEM β-lactamase (TEM). TEM is the enzyme underlying one prominent mechanism of antibiotic resistance in pathogenic Gram-negative bacteria [9]. It confers resistance against β-lactam antibiotics, such as penicillin, by hydrolyzing them into inactive forms. Inhibiting this enzyme is one strategy for restoring the efficacy of β-lactam antibiotics. Current therapies use either mechanism-based inhibitors, such as the natural product clavulanic acid, which irreversibly react with TEM’s active site serine, or transition-state analogs like boronic-acid derivatives [10]. Both types of inhibitors act by sterically blocking the active site, preventing substrate from binding. TEMs that are resistant to these competitive inhibitors have been identified in the clinic, heightening the urgency for new, novel inhibitors that will not be susceptible to pre-existing forms of resistance.

We have previously employed Markov state models (MSMs) of TEM to identify cryptic pockets that are not obvious in the ligand-free crystal structure of TEM [2,11]. An MSM is a network representation of a protein’s energy landscape, consisting of nodes that represent energy minima where the protein tends to dwell and the probabilities of transitioning between these states. They are typically constructed from many independent molecular dynamics simulations and provide a convenient coarse-graining of the data that enables practitioners to quickly identify interesting features. After using MSMs to identify a number of cryptic pockets in TEM [2], we tested them experimentally through a chemical modification technique targeting cysteine residues that become solvent exposed upon pocket opening [11]. There is evidence that small molecules binding in these pockets, either through covalent attachment to the engineered cysteine [11] or non-covalent interactions [7], act as inhibitors.

Here we describe a method for targeting cryptic pockets and apply it to TEM. First, we use docking to screen a library of compounds against a cryptic pocket identified from our MSM. Importantly, instead of targeting a single structure, we employ our recently developed Boltzmann-docking technique [12] to account for conformational heterogeneity in the structure of a pocket. Then we use a high-throughput screen to experimentally test the highest scoring compounds. Finally, we characterize the hit compounds in depth and use site-directed mutagenesis to support our model that they bind in the cryptic pocket as designed. Using this method, we identify one inhibitor and two activator compounds. The inhibitor has an EC50 = 57 ± 3 μM, and while modest relative to TEM inhibitors used clinically, it represents an improvement over inhibitors with *K*_*i*_’s of 500 μM that were found by chance to bind this pocket [7]. While it is not obvious that activators of TEM would be clinically relevant, the ability to design activators could prove useful against other strategically chosen targets. Overall, our results highlight the general utility of our approach for identifying both inhibitors and activator compounds.

## Materials and methods

### Protein structure selection for docking

Structures containing cryptic pockets were selected from our previously constructed MSM for TEM [2]. A Python implementation of LIGSITE [13] was used to identify pocket volume elements within representative structures from each state in this MSM, where the grid step size was set to 1.0 Å and the minimum number of protein-solvent-protein (minPSP) events was set to 6. Contiguous pocket volume elements were grouped together into pockets. Pockets consisting of less than 30 pocket elements (~30 Å^3^) were discarded. For each pocket, the set of structures containing that pocket was identified as follows: 1) the largest unclustered pocket is selected as a new cluster center, 2) all pockets are assigned to the closest cluster center that is within a specified distance cutoff (i.e. if the distance between a pocket’s center of mass and any cluster center’s center of mass is not within the distance cutoff, it remains unassigned), and 3) steps 1–2 are repeated until all pockets are assigned. Representative structures from the 15 most populated states (i.e. populations greater than or equal to 0.004% of the population) that contained the known cryptic pocket were selected as targets for screening (see S1 Dataset). For reference, 80% of the total states in the MSM have populations above this threshold, and it eliminates about half of the states in which the cryptic pocket occurs.

### Small molecule library source and preparation for docking

The compounds used in this work were obtained from “The NCI/DTP Open Chemical Repository” at http://dtp.nci.nih.gov. The database was filtered for compounds that obey Lipinski’s rule of 5 [14], except a molecular weight cutoff of 400 g/mol was used. It was also purged of reactive and promiscuous [15] and aggregation-prone [16] compounds resulting in a total of 12,695 compounds screened. The compounds were all >95% pure as certified by the supplier (NCI DTP Discovery Services) and assumed to be racemic mixtures. The best-predicted binders were ordered from NCI for use in the *in vitro* activity assays. Compounds were dissolved in 100% dimethyl sulfoxide and stored at −20°C. Four compounds could not be solubilized and were not tested.

### Docking

Docking against individual structures was performed with Surflex-dock [17]. The compound structures were generated using the Concord module of SYBYL-X 2.1.1 and minimized using the Tripos force field. Because SMILES strings from the NCI database do not specify stereochemistry, this minimization procedure selects only the lowest energy isomer for docking. Surflex-Dock receptor protomols were generated with a threshold of 0.5 and a bloat of 3.0. These protomols were then used to screen various ligands for receptor complementarity. The Hammerhead scoring function [18] inherent to Surflex was used to score the resulting poses. The default ‘-pgeom’ docking accuracy parameter set was used. Boltzmann-docking scores were then calculated as the weighted-average of the scores for each state, using the equilibrium probabilities of each state as their weights.

### Protein expression and purification

A variant of TEM containing the M182T substitution was used for these studies. The gene was expressed from a pET24 vector (Life Technologies) using an OmpA signal sequence to target it to the periplasm in BL21(DE3) Gold cells (Agilent Technologies).

Cells were induced with 1 mM IPTG at OD = 0.6 and grown at 18°C for 15 h before harvesting. TEM β-lactamases were isolated from the periplasmic fraction using osmotic shock lysis: Cells were resuspended in 30 mM Tris pH 8, 20% sucrose and stirred for 10 min at room temperature. After centrifugation, the pellet was re-suspended in ice-cold 5 mM MgSO_4_ and stirred for 10 min at 4°C. After centrifugation, the supernatant was dialyzed against 20 mM sodium acetate, pH 5.5 and purified using cation exchange chromatography (BioRad UNOsphere Rapid S column) and exchanged into storage buffer (20 mM Tris, pH 8.0) by size exclusion chromatography (BioRad ENrich SEC 70 column).

### Activity assays

A UV-vis plate-based assay was used to experimentally screen compounds identified in the virtual screen. To each well of a 96-well plate was added 1 nM TEM, 2% DMSO, 10% glycerol and 0.01% Triton X-100 in 50 mM potassium phosphate buffer at pH 7.0. Compounds were tested in triplicate at concentrations of 500 nM, 50 μM and 100 μM. The reactions were initiated by addition of 50 μM nitrocefin (Cayman Chemical Company), incubated at 25°C and followed by absorption at 482 nm for 25 seconds using a BioTek Synergy2 Multi-Mode Reader. The enzyme and compound were pre-incubated for 5 minutes prior to addition of substrate. Initial velocities were extracted by fitting the first 10 seconds to a linear equation. We define hits as compounds that had: 1) dose-dependent activity, and 2) an impact greater than or equal to 20% that of the internal control reactions on the same plate containing no compound.

Individual activity assays were performed in a Cary 60 UV-vis spectrometer (Agilent Technologies). For measuring TEM activity, each reaction contained 1–10 nM enzyme, 2% DMSO, 0.01% Triton X-100, 10% glycerol, 10–200 μM nitrocefin and 50–100 μM compound in 50 mM potassium phosphate buffer at pH 7.0. After the enzyme and ligand were incubated for 5 minutes at 25°C, nitrocefin was added and the reaction was followed at 482 nm for 70 seconds. Dose-response curves were acquired at 50 μM nitrocefin. Kinetic parameters (*k*_*cat*_ and *K*_*m*_) were determined by the Michaelis-Menten equation using initial velocity non-linear regression analysis in Kaleidagraph (Synergy Software, v 4.5). Data for compound 1 was fit by a two-parameter activation model (Eq 1), fixing *K*_*m*_ to the value from data taken in the absence of compound, to extract *K*_*act*_ [19].

$$v=\;\frac{k_{cat}\left[E\right]\left[S\right]\left(1+\;\;\frac{\beta \left[A\right]}{K_{act}}\right)}{K_{m}+\left[S\right]\left(1+\;\;\frac{\left[A\right]}{K_{act}}\right)}$$ (1)

Chymotrypsin reactions were followed at 410 nm using 30 nM enzyme and 200 μM N-succinyl-Ala-Ala-Pro-Phe p-nitroanilide at 25°C. β-galactosidase reactions were followed at 420 nm using 4 nM enzyme and 1 mM 2-nitrophenyl β-D-galactopyranoside at 37°C. All assays were performed in triplicate in 50 mM potassium phosphate buffer, 0.01% Triton and 2% DMSO at concentrations of substrate below their *K*_*m*_’s to ensure effects on either *k*_*cat*_ or *K*_*m*_ would be detectable. Enzymes and substrates were purchased from Sigma-Aldrich.

## Results and discussion

### Docking against the crystal structure of a cryptic pocket yields an activator

**Fig 1:**
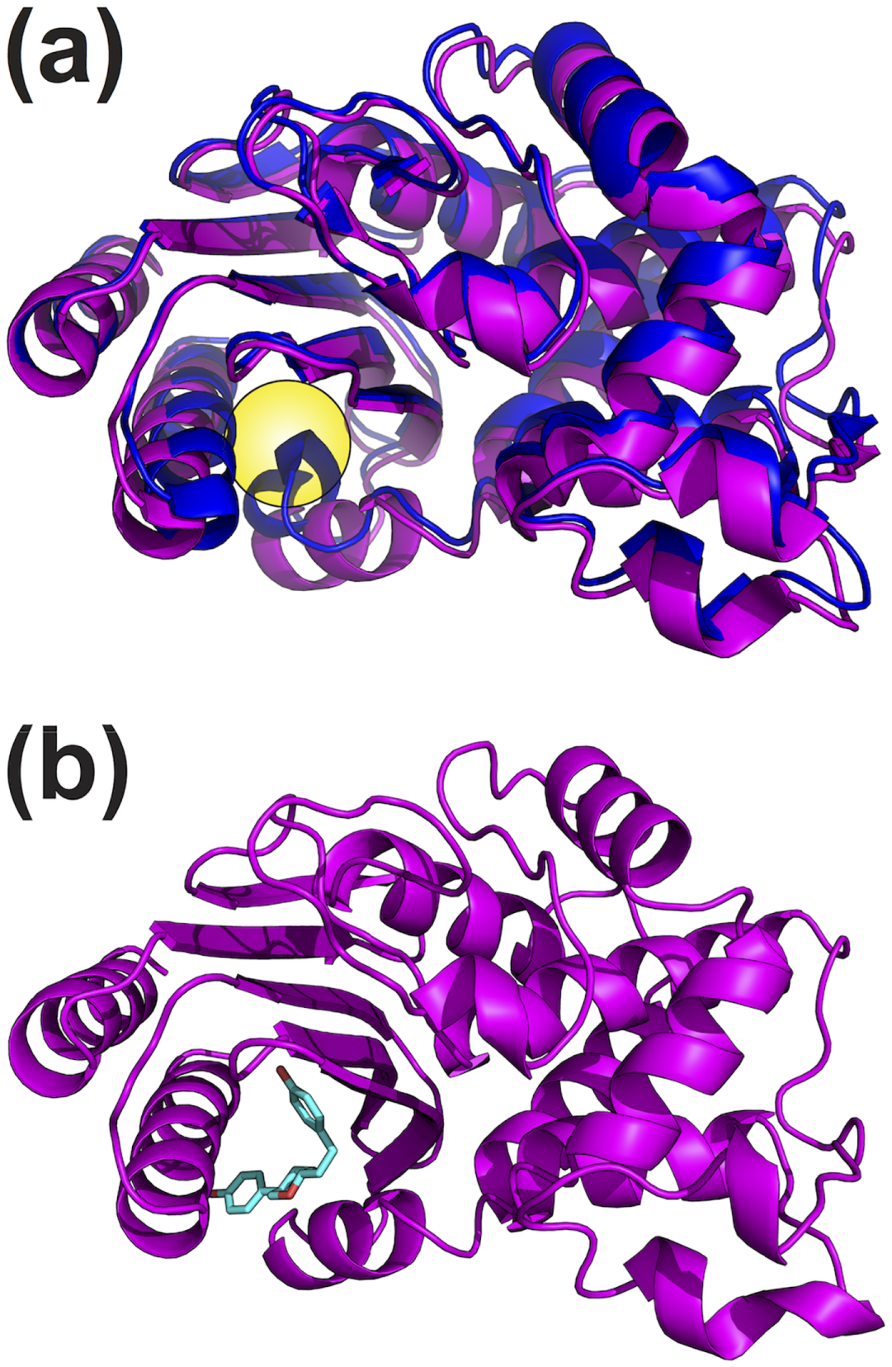
Crystal structure of TEM’s cryptic pocket. (a) Structures of TEM crystallized in the absence of ligands (blue ribbon, 1JWP) and presence of an inhibitor (magenta ribbon, 1PZO), which is removed to highlight its cryptic binding pocket (yellow sphere). (b) A high-scoring, representative pose for compound 1 (cyan sticks).

**Fig 2:**
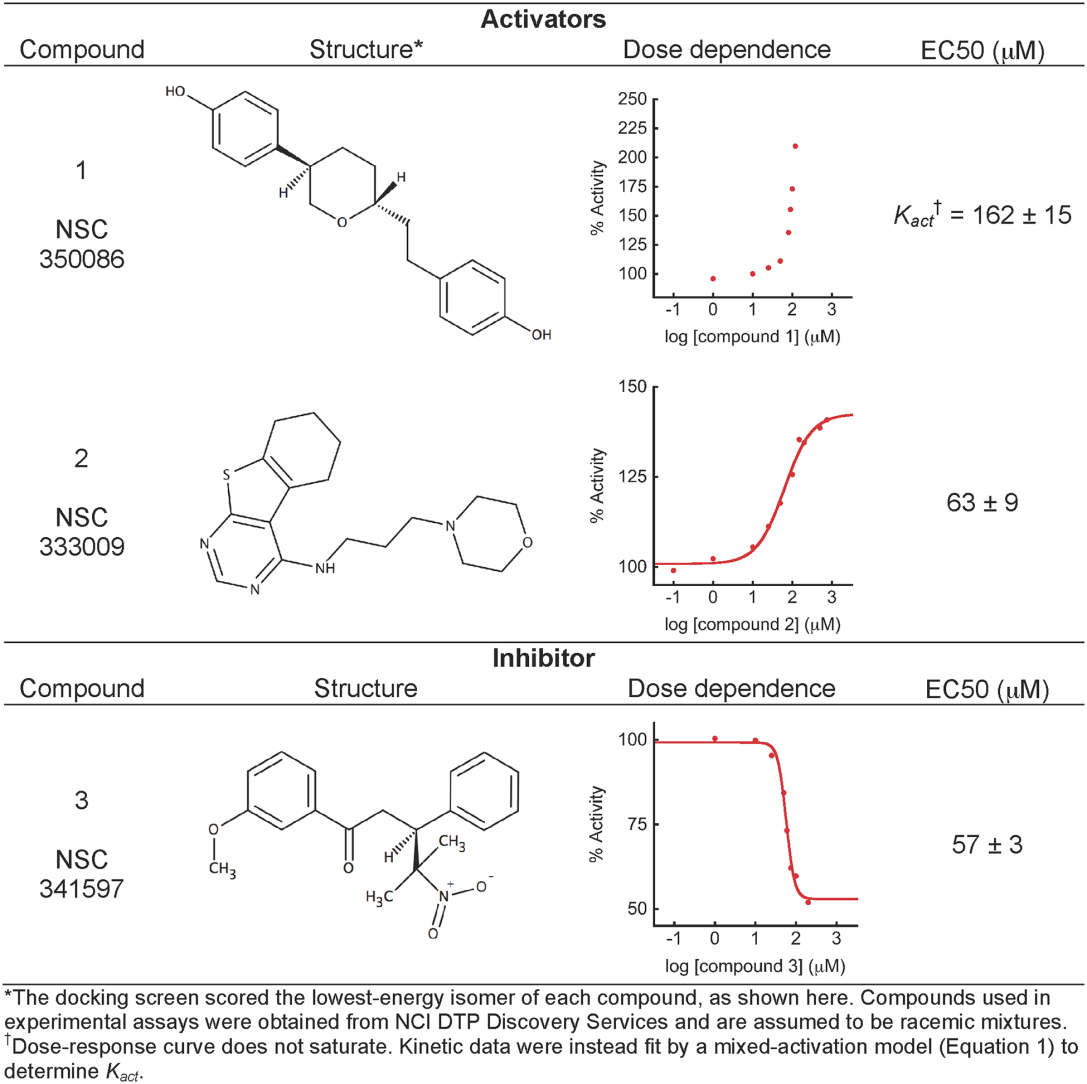
Compound structures, dose-dependence curves and EC50s.

To test our docking protocol and set a baseline for assessing the success of targeting structures from our MSM, we first applied our docking approach to a crystal structure of a cryptic pocket. Only through crystallization of TEM with an inhibitor from a screen was this cryptic pocket revealed [7], as it is not present in the structure when ligand is not bound [20] (Fig 1a). Of the 12,695 compounds we screened *in silico*, we tested the 40 compounds with the highest docking scores for their ability to modulate TEM activity in a high throughput plate-based assay, and identified 5 hit compounds. Of these, 4 were eliminated due to aggregation or non-specific effects (see Methods). The remaining compound 1 (Fig 1b) was further investigated in detail to determine its effect on catalytic efficiencies and its dose dependence (Fig 2). The compound increased TEM’s *k*_*cat*_/*K*_*m*_ for nitrocefin by 52% (S1 Table). Aggregation at high concentration prevented us from obtaining a saturated dose-dependence curve, but fitting initial velocities to a mixed-activation model (Eq 1) results in a dissociation constant for the enzyme-substrate complex, or *K*_*act*_, of 162 ± 15 μM. Although our initial docking screen was performed using only the lowest-energy isomer depicted in Fig 2, our assays contained a racemic mixture. Compound 1 contains two chiral centers, making a total of four possible isomers. We retroactively docked the other three stereoisomers and found that they score similarly, adopting extended conformations to fill the binding pocket (S1 Fig), which suggests they might all contribute to the observed activity. If, however, only one isomer is active, then the dissociation constant reported here likely underestimates the compound’s true affinity. While it may seem surprising to discover an activator during a screen against a pocket previously shown to bind an inhibitor, there is precedent in the literature for compounds to bind to the same location on a protein but have opposite effects on activity [21].

### Docking against an ensemble of cryptic pocket structures yields an activator and an inhibitor

**Fig 3:**
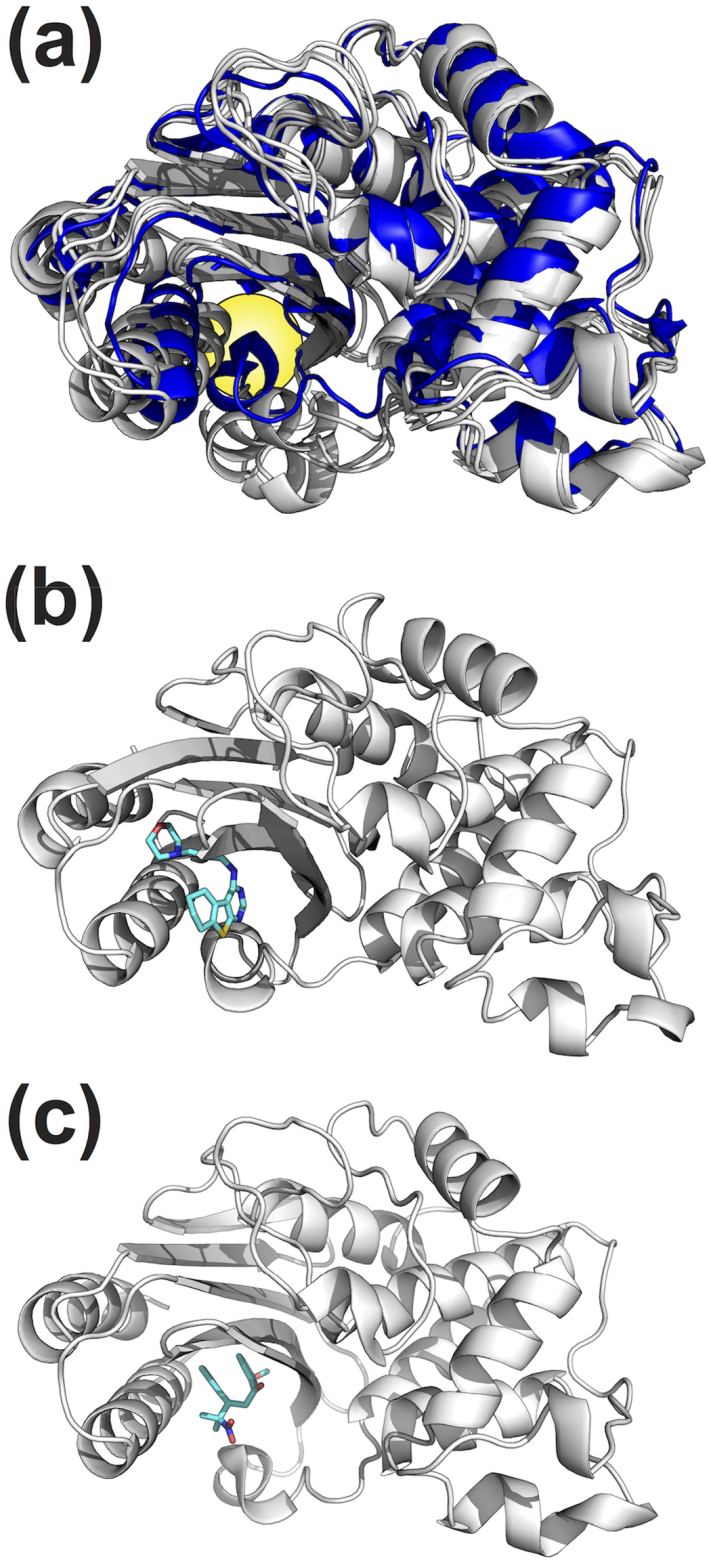
(a) Crystal structure of TEM (blue ribbon, 1JWP) overlaid with 3 representative structures used in Boltzmann docking (white ribbon) to highlight the cryptic pocket (yellow sphere). (b) A high-scoring, representative pose for compound 2 (cyan sticks). (c) A high-scoring, representative pose for compound 3 (cyan sticks).

Having successfully found an activator compound by docking against a crystal structure of the known cryptic pocket, we wanted to test whether docking against an ensemble of pocket structures from our MSM is similarly able to identify novel modulators of TEM activity. Our previous work demonstrated that the cryptic pocket identified in the inhibitor-bound crystal structure is also detectable in simulations of the protein in the absence of compound [2], allowing us to compare our strategies using the same pocket. We docked the same library of compounds described above against 15 structures from the simulation (Fig 3A). These structures were chosen based on two criteria: first, they contained the known cryptic pocket; and second, they are highly populated structures in the MSM (See methods). The most populated state in our set of 15 structures is an order of magnitude more probable than the least populated state, and we reasoned that a compound would require a higher affinity for a lowly populated state than a highly populated state to be an equivalent hit. To account for the differences in populations, we employed a method we developed previously, called Boltzmann docking, to rank the library of compounds. Boltzmann docking takes advantage of population information from the MSM to generate a score that accounts for both interactions between the compound and protein and the probability of the structure being docked against. Previously, we have shown this approach can better predict substrate affinities than docking against single structures [12]. Available compounds with high Boltzmann-weighted docking scores against multiple structures were ordered from the NCI for screening in an *in vitro* plate-based assay. Out of 71 compounds tested, 16 effected TEM activity and were subjected to further testing. Of these, 14 were eliminated due to aggregation or non-specific effects (see Methods). Of the two remaining compounds, one (compound 2, Fig 3b) is an activator, increasing *k*_*cat*_/*K*_*m*_ by 39% with an EC50 of 63 ± 9 μM. The other (compound 3, Fig 3c) is an inhibitor, decreasing *k*_*cat*_/*K*_*m*_ by 59% with an EC50 of 57 ± 3 μM (Fig 2 and S1 Table). Compound 3 has one chiral center, so like compound 1, we docked the alternative isomer for comparison (S1 Fig). The binding pocket accommodates this isomer with a comparable score to our original hit. It is possible, however, that the EC50 we report, which was measured using a racemic mixture, underestimates the active compound’s affinity if one isomer is more active than the other.

We identify hit compounds with similar efficacies whether docking against the crystal structure or structures from the MSM. Although the effects of the three hit compounds are modest compared with true drug molecules with binding affinities in the 60–160 μM range, they are an improvement over inhibitors identified by chance to bind this pocket, which are closer to 500 μM [7]. Furthermore, we find it intriguing that docking against the same pocket yielded both positive and negative modulators. In the future, it would be interesting to understand why the effects of these compounds differ so markedly, with the ultimate objective of being able to predict whether a compound will be an activator or inhibitor.

### The effects of all hit compounds are specific

Many hit compounds identified by virtual or high throughput screening efforts are known to act non-specifically, most commonly via aggregation-based mechanisms [22,23]. We took several precautions at each stage to minimize and identify false positives. At the *in silico* screening level, we filtered the NCI/DTP library for reactive [15] and aggregation-prone [16] compounds. The experimental screens were performed in the presence of 0.01% Triton-X, which is below its critical micelle concentration (0.02%). Our hit compounds were also assayed individually in the absence of Triton-X to test for detergent-dependence, which is consistent with non-specific aggregation-based mechanisms of action. Modulation by compounds 2 and 3 was similar under all conditions (S2 Table). For compound 1, however, no activation was observed in the absence of detergent. This is the opposite effect expected for an aggregation-based mechanism and suggests enhancing the solubility of the compound through addition of 0.01% Triton-X is important for its mode of action, supporting a model for specific binding to TEM. Another hallmark of non-specific aggregation mechanisms is dependence on enzyme concentration [24]. We measured each hit compound’s activity as a function of enzyme concentration and observed the same modulating effects over a ten-fold range of concentrations for compounds 2 and 3. Compound 1 has reduced effectiveness at the highest enzyme concentration tested, so we further investigated potential non-specific effects by testing it against unrelated enzymes. While general mechanisms for non-specific activation have not been thoroughly investigated, it has been suggested that non-specific activators may act like detergents by interfering with adsorption of protein to surfaces [25]. If this were the mechanism for compound 1, or for our other hit compounds, then it should have similar effects on many other types of enzymes. To test this idea, we chose two alternative enzymes with dissimilar structures and activities from TEM, chymotrypsin and β-galactosidase. None of the hits had an impact on the activity of either enzyme (S2 Table), again supporting the idea that the compounds act specifically.

### Eliminating key contacts in the binding pocket abrogates effects

**Fig 4:**
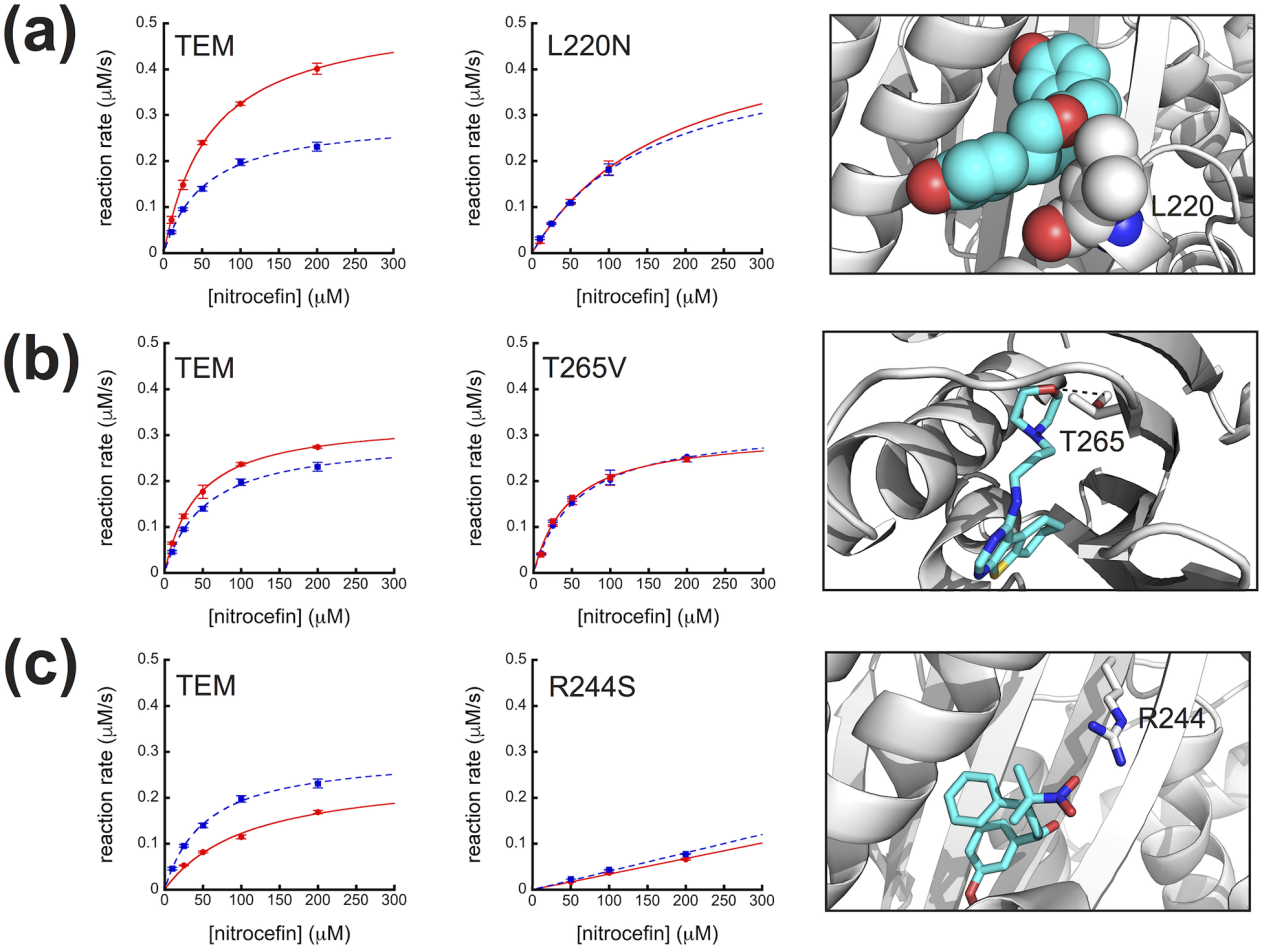
Michaelis-Menten plots for TEM and variants without compound (blue dotted line) and with 100 μM compounds (red solid line) (a) 1, (b) 2 or (c) 3. Error bars are standard deviations. Insets highlight the key contact residues that are substituted in the variants and their interactions with the docked compounds. Compound 1 is shown in cyan spheres to emphasize van der Waals contacts with Leu220, whereas compounds 2 and 3 are shown in cyan sticks to highlight hydrogen bond and electrostatic interactions, respectively. Hydrogen bond is indicated with dashed black line.

We verified that our compounds were binding in the intended pocket by removing key protein contacts, as predicted by our docking models, and measuring activity in the presence and absence of compound (Fig 4). Variants lacking key contacts will have compromised binding affinities, and thus their activities will be less effected by the compounds. Our docking suggests multiple modes of binding for the compounds to the cryptic pocket, due in part to its greasy character. The top ten docking poses for the compounds against all 15 states were evaluated visually to identify recurrent contacts, particularly potential hydrogen bonds and electrostatic interactions that could be targeted by mutagenesis.

For compound 1, all of the sidechains in the cryptic pocket with significant contacts are hydrophobic and form van der Waals interactions with aliphatic regions of the compound. We chose to mutate Leu220 to Asn, which is isosteric but increases the polarity of this residue and thus compromises its ability to interact with compound (Fig 4a). It is also a residue predicted to form significant contacts with each possible isomer (S1 Fig), so we would expect to observe an effect even if multiple isomers are active in our assay. As predicted, activity of the L220N variant is the same both in the presence and absence of compound 1, as demonstrated by the fact that the *k*_*cat*_’s and *K*_*m*_’s measured under both conditions are within error (Fig 4a and S1 Table). This suggests that compound no longer binds in the presence of the L220N substitution. Therefore, we conclude the compound is binding in the cryptic pocket as predicted and that L220 is a key residue for stabilizing the interaction.

To determine whether compounds 2 and 3 were binding in the predicted cryptic pocket, we made variants of TEM lacking key contacts revealed by our docking models (Fig 4b and 4c). Again, most of the sidechains in the cryptic pocket are hydrophobic and form van der Waals interactions with aliphatic regions of the compounds. To minimize perturbation to the protein structure, however, we chose to substitute the residues predicted to form hydrogen bonds or electrostatic interactions with the compounds.

To test the binding of the activator, compound 2, we substituted Thr265 with a Val to eliminate its ability to hydrogen bond to the compound’s morpholinyl oxygen (Fig 4b). Compound 2 increases activity of T265V by 16%, which is about half its effect on TEM without the substitution (S1 Table). While the hydrogen bond clearly contributes to binding, removing it does not completely eliminate binding. Regardless, the reduced efficacy of compound 2 against T265V supports our hypothesis that the activator binds in the cryptic pocket, as intended.

Mutational data also support our hypothesis that the inhibitor, compound 3, binds in the intended pocket. In many of its top poses, and for both isomers (S1 Fig), the negatively charged nitro functional group of the inhibitor, compound 3, is poised to form a favorable electrostatic interaction with Arg244 (Fig 4c). We tested the effect of compound 3 on TEM R244S with the prediction that it would be less able to inhibit the enzyme. Interestingly, Arg244 is a critical residue for substrate binding, and substitutions at position 244 are known to severely compromise activity [26], which suggests a mode of action for our inhibitor. We observe that R244S is much less active than TEM (S1 Table), and the enzyme does not saturate under the conditions of our assay (Fig 4c). Nonetheless, we reasoned that our approach for testing the binding location should apply even in this less active variant, because we can still compare *k*_*cat*_/*K*_*m*_ in the presence and absence of compound 3. Indeed, we observe no inhibition by compound 3 for R244S (S1 Table), suggesting the compound binds in the cryptic pocket and that the electrostatic interaction is formed and important for binding.

## Conclusions

Cryptic pockets are a general feature of many protein folds [2]. Designing drugs that bind them creates opportunities for identifying activator compounds and targeting proteins previously considered “undruggable.” Previously, we demonstrated that a cryptic pocket identified in TEM is also detectable in MSMs of the protein built from molecular dynamics simulations run in the absence of compound [2,11]. Here, we identify small molecule modulators of TEM activity using both crystal structures and conformations from our computational model, showing that either is a suitable target for virtual screening. Out of 111 compounds, we identify 21 that modulate enzyme activity in our high-throughput assay (19% hit rate). After eliminating 18 compounds due to aggregation or non-specific effects, we are left with 3 true hit compounds—two activators and one inhibitor. Mutational analysis suggests these compounds bind in the pocket as designed. Our results suggest it is possible to extend our methodology to many protein targets, even those lacking crystal structures of cryptic pockets.

## Supporting information

S1 Dataset TEM structures against which the library of compounds was docked. (PSE)

S1 Table Michaelis-Menten kinetic parameters for TEM β-lactamase variants with and without compounds 1, 2 and 3. (PDF)

S2 Table Tests for non-specific activity of compounds 1, 2 and 3. (PDF)

S1 Fig Docking alternative isomers of (a) compound 1 and (b) compound 3. Residues targeted in mutagenesis studies are highlighted in (a) spheres or (b) sticks, and docked compounds are shown in cyan. The stereochemistry of each compound is shown below its docked structure.
(TIF)
