# Supplementary material for: Designing small molecules to target cryptic pockets yields both positive and negative allosteric modulators
Source: PLoS One. 2017 Jun 1;12(6):e0178678. doi: 10.1371/journal.pone.0178678 (PMC5453556; doi:10.1371/journal.pone.0178678)
Supplement: S1 Table — (PDF) [file pone.0178678.s002.pdf]

S1 Table. Michaelis-Menten kinetic parameters for TEM β-lactamase variants with and without compounds 1, 2 and 3

	no compound			+ 100 μM compound			% modulation of k_{cat} / K_m
	k_{cat}* (s^{-1})	K_m* (μM)	k_{cat} / K_m ($\mu M^{-1}s^{-1}$)	k_{cat} (s^{-1})	K_m (μM)	k_{cat} / K_m ($\mu M^{-1}s^{-1}$)	
				Compound 1			
**TEM**	295 ± 7	53 ± 3	5.6 ± 0.1	527 ± 8	62 ± 2	8.5 ± 0.1	+52%
**L220N**	4.6 ± 0.6	157 ± 29	3.0 x 10^{-2} ± 0.2	5.2 ± 0.3	183 ± 17	3.0 x 10^{-2} ± 0.1	0%
				Compound 2			
**TEM**	295 ± 7	53 ± 3	5.6 ± 0.1	334 ± 5	43 ± 2	7.8 ± 0.0	+39%
**T265V**	323 ± 10	57 ± 4	5.7 ± 0.1	307 ± 17	47 ± 7	6.6 ± 0.2	+16%
				Compound 3			
**TEM**	295 ± 7	53 ± 3	5.6 ± 0.1	256 ± 28	110 ± 24	2.3 ± 0.3	-59%
**R244S**	ND†	ND†	0.3 ± 0.0	ND†	ND†	0.3 ± 0.0	0%

*Errors are standard errors of the fit.
†Not determined. Michaelis-Menten curve did not saturate. k_{cat}/K_m was determined by a linear fit.
