# Supplementary material for: Designing small molecules to target cryptic pockets yields both positive and negative allosteric modulators
Source: PLoS One. 2017 Jun 1;12(6):e0178678. doi: 10.1371/journal.pone.0178678 (PMC5453556; doi:10.1371/journal.pone.0178678)
Supplement: S2 Table — (PDF) [file pone.0178678.s003.pdf]

S2 Table. Tests for non-specific activity of compounds 1, 2 and 3

	% change in rate		
	**compound 1**	**compound 2**	**compound 3**
**10x enzyme***			
**1 nM TEM**	+65%	+38%	-47%
**10 nM TEM**	+31%	+33%	-48%
**Detergent-dependence***			
**0% Triton-X**	+2%	+30%	-40%
**0.01% Triton-X**	+66%	+33%	-45%
**Alternative enzymes**			
**chymotrypsin**	-5%	-1%	-2%
**β-galactosidase**	+2%	+5%	+3%

*Rates measured at 50 μM nitrocefin.
