# Supplementary figures and images for: Designing small molecules to target cryptic pockets yields both positive and negative allosteric modulators
Source: PLoS One. 2017 Jun 1;12(6):e0178678. doi: 10.1371/journal.pone.0178678 (PMC5453556; doi:10.1371/journal.pone.0178678)

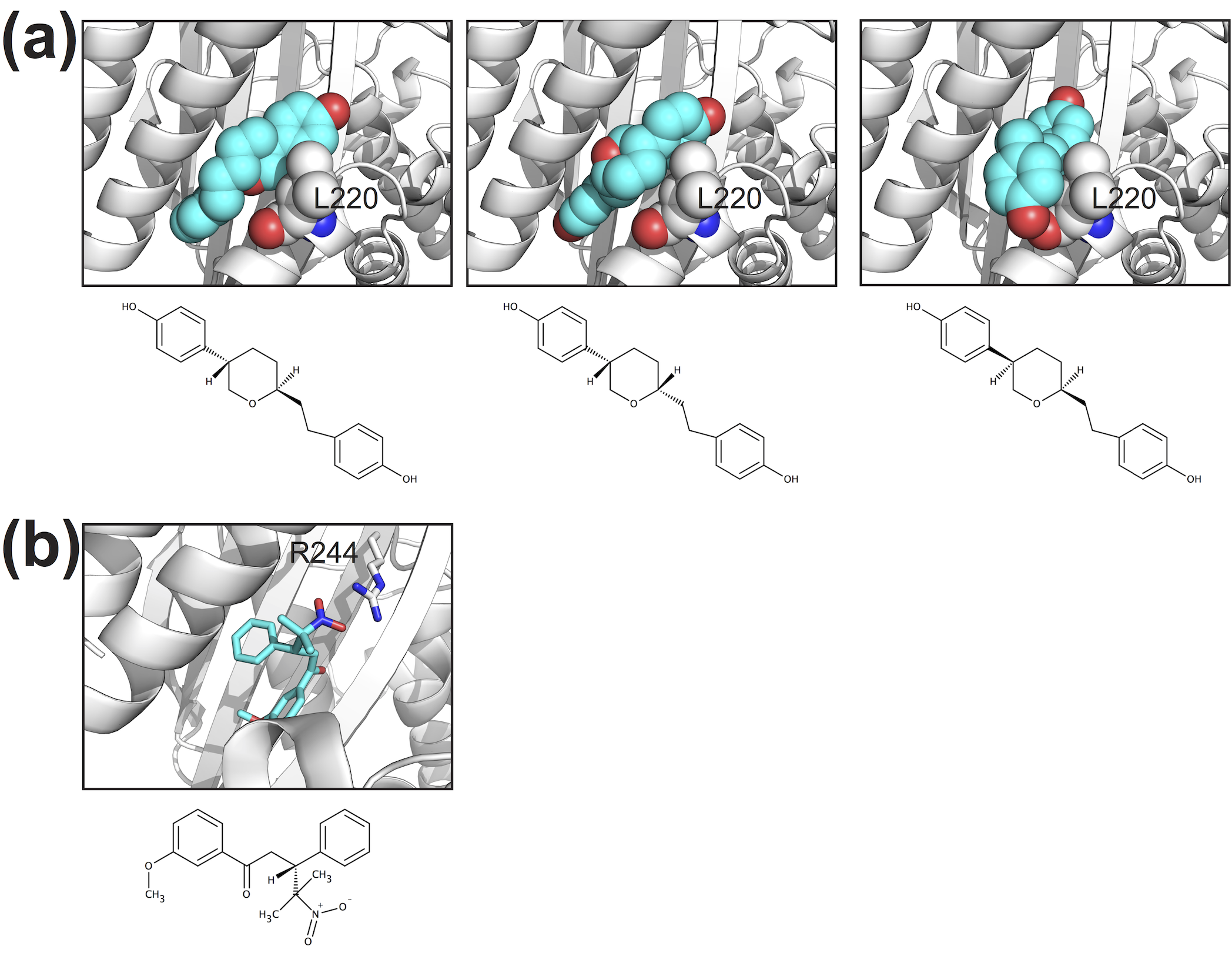

Supplement: S1 Fig — Residues targeted in mutagenesis studies are highlighted in (a) spheres or (b) sticks, and docked compounds are shown in cyan. The stereochemistry of each compound is shown below its docked structure. (TIF) [file pone.0178678.s004.tif]
